# Weekly symptom profiles of nonhospitalized individuals infected with SARS‐CoV‐2 during the Omicron outbreak in Hong Kong: A retrospective observational study from a telemedicine center

**DOI:** 10.1002/jmv.28447

**Published:** 2023-01-09

**Authors:** Jingyuan Luo, Jialing Zhang, Hiu To Tang, Hoi Ki Wong, Yanfang Ma, Duoli Xie, Bo Peng, Aiping Lyu, Chun Hoi Cheung, Zhaoxiang Bian

**Affiliations:** ^1^ Hong Kong Chinese Medicine Clinical Study Centre, School of Chinese Medicine Hong Kong Baptist University Hong Kong SAR China; ^2^ Centre for Chinese Herbal Medicine Drug Development Hong Kong Baptist University Hong Kong SAR China; ^3^ School of Chinese Medicine Hong Kong Baptist University Hong Kong SAR China

**Keywords:** COVID‐19, Omicron, symptom profile

## Abstract

Omicron BA.2.2 is the dominant variant in the Hong Kong outbreak since December 31, 2021. There is no study reporting the weekly symptom profile after infection. In this retrospective study, participants who tested positive for SARS‐CoV‐2 after December 31, 2021, and registered in the telemedicine system between March 14 and May 6, 2022, were analyzed. Among registered 12 950 self‐quarantined COVID‐19‐positive patients, 11 776 symptomatic patients were included for weekly symptom profile analysis. A total of 4718 (40.1%) patients reported symptoms in the first week after a positive test, 2501 (21.2%) in the second week, 1498 (12.7%) in the third week, 1048 (8.9%) in the fourth week, and 2011 (17.1%) in over 4 weeks. Cough was the most common symptom in all participants. Patients in the first week had higher odds of reporting fever (0.206, 95% confidence interval [CI]: 0.161–0.263, *p* < 0.001) and sore throat (0.228, 95% CI: 0.208–0.252, *p* < 0.001). Patients in over 4 weeks had higher odds of reporting fatigue (1.263, 95% CI: 1.139–1.402, *p* < 0.001). Further, having at least two vaccine doses linked to lower odds of having fever (0.675, 95% CI: 0.562–0.811, *p* < 0.001), but not associated with the presence of cough and fatigue. Diabetic patients had higher odds of reporting diarrhea (1.637, 95% CI: 1.351–1.982, *p* < 0.001). Symptoms from Omicron infection may last for more than 4 weeks and symptom profiles vary from week to week. Vaccination and comorbidity affect the symptom profiles.

## 1 INTRODUCTION

Several variants of severe acute respiratory syndrome coronavirus 2 (SARS‐CoV‐2) have caused large outbreaks of infection and mass mortality since early 2020. In November 2021, WHO designated a SARS‐CoV‐2 variant, first detected in Botswana and South Africa, and spreading globally, as “omicron.”^1^ Community outbreaks of the Omicron variant began around the end of 2021, with Omicron BA.2.2 eventually becoming the largest wave of the pandemic so far.^2^ There were over 1.2 million reported SARS‐Cov‐2 infection cases (by nucleic acid tests or rapid antigen tests [RATs]) and 9183 deaths in Hong Kong from December 31, 2021, to July 1, 2022.^3^

Symptoms characteristic of COVID‐19 include respiratory symptoms, fever, gastrointestinal symptoms, and neurological issues.^4^, ^5^, ^6^ But symptom profiles vary according to the virus variant,^7^, ^8^ culture and geographic location,^9^ comorbidities,^9^ and duration of illness.^10^ Several studies on the characteristics of the Omicron variant indicate that it causes less severe illness compared to previous variants.^11^, ^12^, ^13^, ^14^, ^15^ A large prospective observational study (*n* = 63 002) in the United Kindom in early 2022 comparing Omicron and Delta infections revealed that respiratory and systemic symptoms were high in both within 21 days after being infected, but other symptoms, except sore throat and hoarse voice, were less severe with Omicron.^15^ The average duration of symptoms among Omicron cases was 6.87 days (95% confidence interval [CI]: 6.58–7.16) which is shorter than Delta cases (mean duration 8.89 days, 95% CI: 8.61–9.17).^15^ A small retrospective study (*n* = 107) of the characteristics of Omicron infection in children (<18 years old) reported that the most prevalent symptom was fever (73.1%).^16^ There are no detailed published reports on the prevalence and severity of Omicron‐induced symptoms in different weeks of illness.

According to the quarantine policy in Hong Kong, persons infected with COVID‐19 or in close contact should be quarantined, regardless of the presence or absence of symptoms.^17^ In addition, a series of stringent social distancing measures were announced by the government during the 5th wave of the outbreak.^3^ Under such circumstances, many infected and quarantined patients sought medical service via the telemedicine center of Hong Kong Baptist University.^18^ This study aims to investigate the prevalence and severity of symptoms in different weeks of COVID‐19 disease and explored their associations by adjusting for age, gender, vaccination status, and comorbidity in a large COVID‐19‐infected population from Hong Kong during a period of Omicron variant dominance.

## 2 METHODS

### 2.1 Study design and participants

Patients who were diagnosed with COVID‐19 with a positive polymerase chain reaction (PCR) test or RAT for SARS‐CoV‐2 by either throat or nose swab after December 31, 2021, and who had a consultation at the HKBU Tele‐Chinese Medicine Centre (HKBU‐TCMC) from March 14 to May 6, 2022, were eligible to participate. PCR and RAT can only be references for the diagnosis of COVID‐19,^19^ but negative results of RAT or PCR were crucial records for quarantined patients to be discharged from isolation.^20^ Chinese medicine physicians communicated with the registered participants via WhatsApp and recorded the demographic information, date of the first positive test of PCR or RAT, date of the first negative test, comorbidities, vaccination history, clinical symptoms and signs, and herbal formulas prescribed for patients in the telemedicine system during the consultation. Fixed symptoms and signs in the system included respiratory symptoms (cough, sputum, itchy throat, sore throat, dry throat, and chest tightness), digestive symptoms (diarrhea, abdominal distension, abdominal pain, and nausea), and systemic symptoms (fatigue, headache, muscle pain, fever, and chill). The definitions of fixed symptoms and signs were listed in Supporting Information: Table 1. Besides, other symptoms could be input by Chinese medicine physicians in a text box. The severity of fixed symptoms was recorded on a 6‐point scale (0–5) with a higher score indicating greater severity, and 0 representing no symptom. Any person who tested positive during the 5th COVID‐19 outbreak in Hong Kong (starting from December 31, 2021) could register for this service. There was no limit to the number of days after being diagnosed. After the consultation, the prescribed Chinese medicine will be delivered to the quarantined venue. No charge for the whole service.

### 2.2 Ethics

All patients provided informed consent at the start of the online consultation in HKBU‐TCMC. All personal data were de‐identified. As this retrospective study used anonymized data, no ethical approval was sought. The reporting of this study followed the recommendations of the STROBE (Strengthening the Reporting of Observational Studies in Epidemiology) guideline.^17^

### 2.3 Statistical methods

The statistical analyses were performed using the Statistical Packages of Social Sciences for Windows (SPSS; version 27.0). Statistical significance was defined as a two‐sided *p*‐value <0.05. The clinically significant for symptom prevalence was defined as: (i) the absolute difference between comparison and control groups is ≥10%; (ii) the ratio of compared figures is greater than 2 times or less than 0.5 times; (iii) the clinically significant for symptom severity is defined as the absolute difference of symptom severity scores between two groups is greater than 1 point. Normally distributed continuous variables are reported as mean (SD) or 95% CI; nonnormally distributed continuous variables are described in terms of the median (interquartile range [IQR]). The differences in continuous variables among patients in different weeks were assessed using the analysis of variance or Kruskal–Wallis nonparametric test. The differences in continuous variables between the two groups were assessed by Student's *t*‐test or nonparametric Mann–Whitney *U*‐test. As for categorical variables, *χ* ^2^ test was used. Correlation analyses were performed using a multivariable logistic regression model for prevalence and a linear regression model for symptom severity. Association is presented by the adjusted odd ratio (AOR). Both were adjusted for age, sex, days after being diagnosed with COVID‐19 (days from the date of first positive PCR or RAT test result to the date of having consultation), comorbidities (chronic diseases), vaccination status (incomplete vaccination was considered to be none or one dose of vaccine; complete vaccination was considered to be at least two doses of vaccine).

## 3 RESULTS

### 3.1 Demographic and clinical characteristics of study population

**Figure 1:**
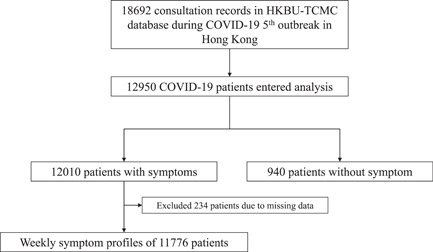
Analysis flowchart of 12 950 COVID‐19 patients

HKBU‐TCMC database has 18 692 consultation records between March 14 and May 6, 2022, during the 5th wave of the COVID‐19 outbreak dominated by the Omicron variant. After excluding nonfirst consultation records, a total of 12 950 self‐quarantined but not hospitalized COVID‐19 patients (with positive PCR or RAT) were included in this study (Figure 1). The median age of included patients was 47 years (IQR: 35–61), 68.0% of patients were female, 62.3% of patients had at least two doses of vaccine, and 23.6% of patients with at least one comorbidity. The median number of days from the first positive test (PCR/RAT) to registration was 11 (IQR: 5–24). The demographic and clinical characteristics of the included patients were listed in Table 1. Of the included patients, 12 010 (92.7%) reported at least one type of COVID‐19 symptom and 940 (7.3%) reported no COVID‐19 symptoms at the time of consultation, which could be asymptomatic or already recovered. Among 12 010 symptomatic patients, 234 (2.0%) patients were excluded from the weekly symptom profile analysis as their first positive test dates were not recorded in the system.

**Table 1:** Clinical characteristics of the study patients, according to absence or presence of at least one COVID‐19 symptom

Characteristic	Total (*N* = 12 950)	Without COVID‐19 symptoms (*N* = 940)	With COVID‐19 symptoms (*N* = 12 010)
Age (years)	47 (35–61)	45 (31–60)	47 (36–61)
Gender (*N*, %)			
Male	4147 (32.0)	327 (34.8)	3820 (31.8)
Female	8803 (68.0)	613 (65.2)	8190 (68.2)
Dose of vaccination (*N*, %)			
Incomplete vaccination	4482 (37.7)	368 (39.1)	4514 (37.6)
Complete vaccination	8068 (62.3)	572 (60.9)	7496 (62.4)
Days between last vaccination to infection	57 (20, 180)	57 (22, 173)	57 (20, 180)
Comorbidities (*N*, %)			
No comorbidity	9895 (76.4)	763 (81.2)	9132 (76.0)
1 or more comorbidities	3055 (23.6)	177 (18.8)	2878 (24.0)
Subgroup of comorbidity (*N*, %)			
Hypertension	1833 (14.2)	109 (11.6)	1724 (14.4)
Diabetes	945 (7.3)	52 (5.5)	893 (7.4)
Hyperlipidemia	1032 (8.0)	52 (5.5)	980 (8.2)
Respiratory disease	159 (1.2)	13 (1.4)	146 (1.2)
Cancer	153 (1.2)	11 (1.2)	142 (1.2)
Cardiovascular disease	373 (2.9)	24 (2.6)	349 (2.9)
Liver/renal disease	135 (1.0)	10 (1.1)	125 (1.0)
Days after being diagnosed with COVID‐19	11 (5, 24)	26 (12, 40)	10 (5, 22)
Days from first positive COVID‐19 test to first negative test	8 (7–10)	8 (7–10)	8 (7–10)
Number of cases at different weeks after being diagnosed with COVID‐19 (days)			
≤7	4855 (37.5)	137 (15.1)	4718 (40.1)
8–14	2633 (20.3)	132 (14.6)	2501 (21.2)
15–21	1620 (12.5)	122 (13.5)	1498 (12.7)
21–28	1142 (8.8)	94 (10.4)	1048 (8.9)
>28	2432 (18.8)	421 (46.5)	2011 (17.1)
Missing	268 (2.1)	34 (3.6)	234 (2.0)

*Note*: Data are presented as medians (IQR) or number (%).

Abbreviation: IQR, interquartile range.

### 3.2 Weekly symptom profiles

**Figure 2:**
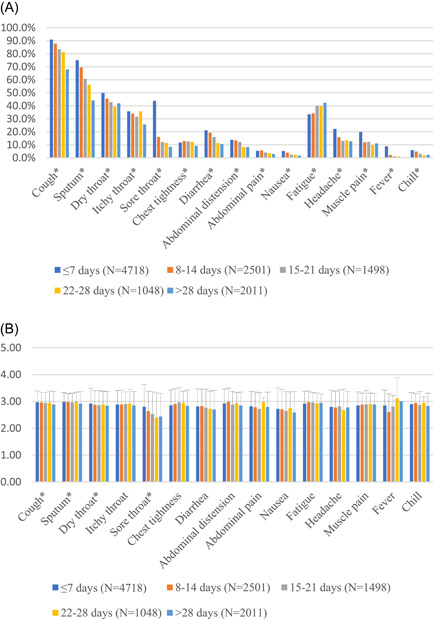
Symptom profiles at different weeks after COVID‐19 diagnosis. (A) Prevalence of symptoms represented as percentage, (B) severity scores of symptoms represented as mean (SD). *Significant difference (*p* < 0.05).

Among 11 776 symptomatic patients, a total of 4718 (40.1%) patients reported symptoms in the consultation within the first week after their first positive COVID‐19 test. The common symptoms (“common” defined as >20% prevalence) in the first week were cough (90.9%), sputum (75.1%), dry throat (49.9%), sore throat (43.8%), itchy throat (35.8%), fatigue (33.5%), headache (22.2%), and diarrhea (21.2%). In the second week of the positive test, 2501 (21.2%) patients reported symptoms, the common symptoms were cough (87.8%), sputum (69.5%), dry throat (45.5%), fatigue (34.2%), and itchy throat (34.0%). In the third week of the positive test, 1498 (12.7%) patients reported symptoms, the common symptoms included cough (83.4%), sputum (60.7%), dry throat (42.8%), fatigue (39.9%), and itchy throat (31.6%). In the fourth week of the positive test, 1048 (8.9%) patients reported symptoms and the common symptoms were cough (81.3%), sputum (56.2%), fatigue (39.8%), dry throat (39.3%), and itchy throat (35.7%). There were 2011 (17.1%) patients who reported symptoms in over 4 weeks, the common symptoms were cough (68%), sputum (44.1%), fatigue (42.4%), dry throat (41.9%), and itchy throat (25.7%) (Figure 2). Symptom prevalence profiles in different weeks after being diagnosed with COVID‐19 were significantly different (*p* < 0.01).

There were five common symptoms in all participants: cough, sputum, dry throat, itchy throat, and fatigue. Sore throat (43.8%), headache (22.2%), and diarrhea (21.2%) were only common in the first week of being positive. Cough and sputum were the first and second most common symptoms in all periods. Apart from these two symptoms, the most common symptom in the first 3 weeks was dry throat. But starting from the fourth week, fatigue became the third most common symptom. Apart from fatigue, the prevalence of all symptoms decreased as the days increased. Notably, sore throat was common in the first week, 2.7 times higher than that in the second week (43.8% vs. 16.1%, *p* < 0.0001), and 4.6 times higher than after 4 weeks or more (43.8% vs. 8.4%, *p* < 0.0001). Fever was more common in the first week, 4 times higher than in the second week (8.9% vs. 2.2%, *p* < 0.0001), and over 20 times higher than after 4 weeks or more (8.9% vs. 0.4%, *p* < 0.0001), but among some patients, fever can last for 2 weeks (2.2%) to 4 weeks (0.4%). The severity level (0–5 points) of all symptoms ranged between 2 and 3 points (Figure 2). Cough, sputum, dry throat, and sore throat had a significant higher scores in the first week of the illness than other weeks (*p* < 0.05). However, the differences in scores for each symptom were not clinically significant (within 1 point).

**Figure 3:**
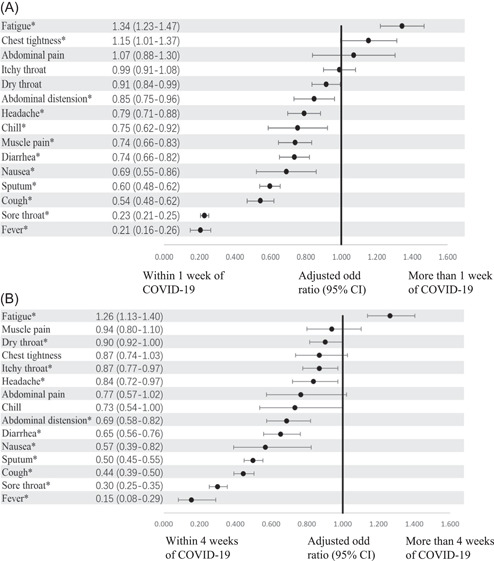
Association between presence of symptoms and different weeks of symptomatic COVID‐19. (A) Adjusted odd ratio of presenting symptoms at the first week of COVID‐19 versus beyond the first week. (B) Adjusted odd ratio of presenting symptoms for ≥4 weeks of COVID‐19 versus <4 weeks. Error bars indicate 95% CI. *AOR that are statistically significant (*p* < 0.05). AOR, adjusted odd ratio; CI, confidence interval.

Patients in the first week of illness had the significant higher odds of reporting cough, sputum, sore throat, diarrhea, abdominal distension, nausea, headache, muscle pain, fever, and chills than other weeks (*p* < 0.05) (Figure 3). Fever (AOR: 0.206, 95% CI: 0.161–0.263, *p* < 0.001) and sore throat (AOR: 0.228, 95% CI: 0.208–0.252, *p* < 0.001) were the most distinguishing symptoms in the first week (Figure 3). Fatigue (AOR: 1.263, 95% CI: 1.139–1.402, *p* < 0.001) was the only symptom that was significantly associated with over 4 weeks of illness (Figure 3).

### 3.3 Association between symptom profiles and the risk factors

**Figure 4:**
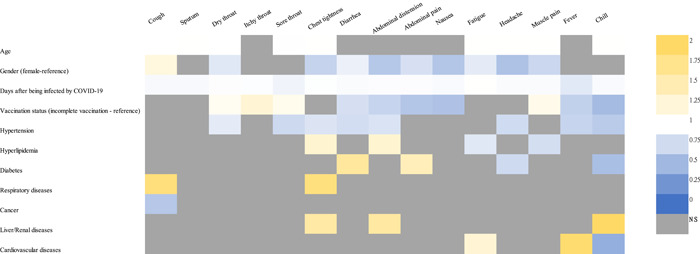
Logistic regression matrix for the association between COVID‐19 symptoms and risk factors (age, gender, days after being diagnosed, vaccination status, and underlying chronic disease). Each column represents the AOR of the different factors in a multivariable model for the presence of the symptom corresponding to the column. Odds ratio which are statistically significant (*p* < 0.05) are colored; not significant OR are marked as NS. AOR, adjusted odd ratio.

The association between each of the symptoms and the risk factors were analyzed by a multivariable logistic regression model, and the data are presented in Figure 4. Fully vaccinated (at least two vaccine doses) had a significantly lower chance with fever (AOR: 0.675, 95% CI: 0.562–0.811, *p* < 0.001), chills (AOR: 0.521, 95% CI: 0.435–0.624, *p* < 0.001), and digestive symptoms include diarrhea (AOR: 0.778, 95% CI: 0.705–0.858, *p* < 0.001), abdominal distension (AOR: 0.697, 95% CI: 0.623–0.781, *p* < 0.001), abdominal pain (AOR: 0.601, 95% CI: 0.506–0.714, *p* < 0.001) and nausea (AOR: 0.574, 95% CI: 0.473–0.696, *p* < 0.001), but higher odds of reporting itchy throat (AOR: 1.278, 95% CI: 1.178–1.386, *p* < 0.001), sore throat (AOR: 1.120, 95% CI: 1.022–1.227, *p* = 0.015), and muscle pain (AOR: 1.139, 95% CI: 1.023–1.269, *p* = 0.018). Interestingly, there is no significant association between vaccination status and the prevalence of cough, sputum, chest tightness, fatigue, and headache (*p* > 0.05).

Patients with diabetes had higher odds with diarrhea (AOR: 1.637, 95% CI: 1.351–1.982, *p* < 0.001) and abdominal pain (AOR: 1.460, 95% CI: 1.036–2.058, *p* = 0.031). Patients with respiratory diseases had the greatest odds of cough (AOR: 1.864, 95% CI: 1.035–3.357, *p* = 0.038) and chest tightness (AOR: 1.840, 95% CI: 1.203–2.816, *p* = 0.005). The chances of abdominal distension were higher among patients with hyperlipidemia (AOR: 1.308, 95% CI: 1.049–1.630, *p* = 0.017) and liver or renal diseases (AOR: 1.610, 95% CI: 1.042–2.489, *p* = 0.032). The chances of fatigue (AOR: 1.287, 95% CI: 1.029–1.611, *p* = 0.027) and fever (AOR: 1.944, 95% CI: 1.229–3.076, *p* = 0.005) were higher for patients with cardiovascular diseases. Patients with liver or renal diseases had higher odds for reporting chills (AOR: 2.036, 95% CI: 1.107–3.746, *p* = 0.022).

AORs for patients in different age in reporting those 15 symptoms were around 1, which indicates the chances of developing those symptoms were not affected by linearly increasing age. Apart from cough (AOR: 1.218, 95% CI: 1.086–1.365, *p* = 0.001), male patients had lower odds (AOR < 1) than female patients for reporting the other symptoms (*p* < 0.05). Symptom profiles by age, gender, vaccination status, and comorbidity were presented in Supporting Information: Figure 1. These risk factors can also affect the severity scores for each symptom; however, differences of scores for each symptom were not clinically significant. A multivariable linear regression matrix of symptom severity scores and risk factors was presented in Supporting Information: Figure 2.

### 3.4 The characteristics between symptomatic and asymptomatic patients within 7 days after testing positive

There was no statistically significant difference (*p* > 0.05) of the baseline characteristics between asymptomatic and symptomatic patients, which included age (45 [IQR: 24.5–64] vs. 45 [IQR: 34–61], *p* = 0.066), gender (female: 64.2% vs. 66.9%, *p* = 0.508), vaccination status (complete vaccination: 51.8% vs. 60.0%, *p* = 0.055), comorbidities (no comorbidity: 17.5% vs. 21.1%, *p* = 0.309), and days between last vaccination to infection between two groups (40.5 [IQR: 22.5–87.75] vs. 48 [IQR: 20–173], *p* = 0.753) (Supporting Information: Table 2). The results from the logistic analysis showed age, gender, vaccination status, with or without comorbidity, and days from testing positive to the consultation were not associated (*p* > 0.05) with the likelihood of being asymptomatic or asymptomatic COVID‐19 patients within 7 days of infection (Supporting Information: Table 3).

## 4 DISCUSSION

To our knowledge, this study provides for the first time detailed weekly symptom profiles of patients with COVID‐19 during the Omicron‐dominated outbreak. We analyzed data from 12 950 patients who tested positive by PCR or RAT for SARS‐CoV‐2 and who sought Chinese medicine consultation and treatment from the HKBU‐TCMC in Hong Kong, from March to May 2022, during the Omicron‐dominant wave of the COVID pandemic. In symptomatic patients, a total of 40.1% patients had symptoms within the first week of tested positive, and 17.1% patients had symptoms even over 4 weeks after the first positive SARS‐CoV‐2 test. A prospective study in the UK reports the symptom duration in Omicron variants is around a week.^15^ In this study, a significant percentage of patients reported symptoms more than 1 week after being diagnosed as positive. This suggests Omicron may have longer effects on patients, which could increase stress for public medical resources. As for the influence after 4 weeks, more investigation work is needed to seek whether and how these patients have more symptoms and signs.

The first and second most common symptoms within 4 weeks were cough and sputum. The third most common symptom was dry throat in the first 3 weeks, and it became fatigue from the fourth week of illness. But the prevalence of fever was low. Symptoms induced by the ancestral SARS‐CoV‐2 strain were predominantly fever.^5^, ^6^ Less prevalence of fever in the Omicron outbreak may be because the duration of fever was short and had generally subsided before the patient's online consultation. For the first time, the severity scores for each symptom were reported, and the average scores indicate a mild to moderate severity of symptoms within 4 weeks. It indicates the severity of Omicron‐induced symptoms for those nonhospitalized quarantined patients was not serious.

Fever and sore throat were shown to be the most distinguishable symptoms for the first week, while patients who had COVID‐19 for more than 4 weeks were significantly more likely to have fatigue. It is consistent with the findings from the previous study of predictors of long COVID.^10^ It should be noted that long COVID can involve multiple organs and can affect many systems including, but not limited to, the respiratory, cardiovascular, neurological, gastrointestinal, and musculoskeletal systems. The symptoms of long COVID include fatigue, dyspnea, cardiac abnormalities, cognitive impairment, sleep disturbances, symptoms of posttraumatic stress disorder, muscle pain, concentration problems, and headache.^21^ Future study needs to focus on long COVID induced by Omicron infection.

The results represent clinical evidence of the association of the prevalence of symptoms and risk factors including age, gender, vaccination status, and comorbidity. Age is not linearly associated with developing symptoms. Studies of other variants of SARS‐Cov‐2 report that greater age is a risk factor of more and more severe symptoms.^22^ Our study also found that apart from cough, female patients were more likely to suffer from the majority of other symptoms, which is consistent with other studies.^23^ Besides, patients with different underlying comorbidities had different odds of displaying specific symptoms. This is consistent with findings from a global study.^9^ It is worth noting that patients with diabetes had the greatest chances of digestive symptoms such as diarrhea and abdominal distension. The mechanism behind this correlation is unclear and deserves further study.^24^

Furthermore, vaccination status, complete or incomplete, is not associated with being asymptomatic or symptomatic patients. There is no significant association between vaccination status and the prevalence of cough, sputum, chest tightness, fatigue, and headache. However, patients with complete vaccination had lower chances with fever, chills, and all digestive symptoms but higher chances for the itchy throat, sore throat, and muscle pain. A study of population immunity to the severe Omicron BA.2 outbreak in Hong Kong suggested that the seroprevalence of neutralizing antibodies is much lower than that against other variants.^25^ Besides, Omicron variants are more resistant to antibodies from vaccinated patients.^26^ This could be the reason why complete vaccination cannot prevent COVID‐19 patients from developing symptoms and exhibiting some symptoms. Nevertheless, vaccination can reduce some symptoms of Omicron,^27^ which is consistent with our findings.

All patients in this study were Chinese in Hong Kong. A study suggested that health‐seeking behaviors in Hong Kong Chinese population are varied by age and symptoms, older adults who had fever, sore throat, and headache were more likely to seek consultation.^28^ Besides, treatment policy in local government may also affect the symptom prevalence. During the 5th outbreak in Hong Kong, treatment strategies include supportive therapy, symptomatic treatment, antiviral agents, and Chinese medicine.^29^ The antiviral drugs (Paxlovid and Molnupiravir) were available for prescription to reduce the risk of death and progression to severe COVID‐19 for high‐risk patients in Hong Kong from February 26, 2022 (Molnupiravir), and March 16, 2022 (Paxlovid).^30^, ^31^, ^32^ However, due to the overwhelming number of positive cases and limited medical resources, according to clinical management guidelines for COVID‐19 from the Hong Kong Hospital Authority,^33^ only limited patients (in‐hospitalized or high‐risk) were treated with these two antiviral drugs. The effectiveness of Paxlovid and Molnupiravir on reducing risks of death and disease progression have been reported in two observational studies,^30^, ^31^ but the effect of these two drugs on symptoms were still unknown, which needs further study.

Strengths of our study include (1) large sample size; (2) the first report of Omicron‐induced symptom profiles (both prevalence and severity scores) in different periods of illness; (3) professional collection of data (i.e., not self‐reported); (4) use of telemedicine which can reach many more patients than face‐to‐face medical consultations; (5) standardized formatting of data and online data collection.

Our study has several limitations: (1) The ratio of symptomatic patients and asymptomatic patients in this study may not reflect the real‐world situation as selection bias exists in patients who seeking online consultation are more likely to have symptoms; (2) although an electronic system that can standardize the data formatting and data collection process, only 15 fixed symptoms were selected for facilitating Chinese Medicine treatment. Other COVID‐19‐related symptoms like loss of sense of smell and taste were not included in this study; (3) the positive PCR or RAT and other medical histories of each subject was according to their self‐reported (uploaded photos of positive results), not from any surveillance system; (4) symptoms and signs can be influenced by multiple factors that had not been recorded, such as medication therapy, previous infection of COIVD, occupation, lifestyle factors, co‐infections by other viruses or bacteria, or allergy. These unknown confounding factors may have biased our findings.

In summary, as it is known that the mutations in the SARS‐CoV‐2 Omicron virus have increased its transmissibility,^34^ there is very limited, specific information on disease progression in those infected with the Omicron variant.^35^ Therefore, it is important to characterize the symptoms features in different periods of illness and improve the accuracy of prognosis and targeted management of Omicron infection, increase the efficiency of health care systems clinicians, and facilitate further research about the effect of mutations in future variants. Importantly, this study provides evidence for a future study about Omicron‐induced long COVID. Furthermore, during the COVID pandemic, telemedicine mode has been playing a major role in providing efficient, widespread medical service. Combining telemedicine with big data analysis will provide much useful evidence for medical research.

## AUTHOR CONTRIBUTIONS

Jingyuan Luo and Jialing Zhang contributed equally to this study. Zhaoxiang Bian developed the concept of this study, and Zhaoxiang Bian and Chun Hoi Cheung led the study. Zhaoxiang Bian, Jingyuan Luo, Jialing Zhang, Chun Hoi Cheung, Duoli Xie, Bo Peng, and Aiping Lyu were responsible for conceptualization. Zhaoxiang Bian, Jingyuan Luo, and Jialing Zhang did data curation. Jingyuan Luo, Jialing Zhang, Yanfang Ma, Hoi Ki Wong, and Hiu To Tang were responsible for the formal analysis. Jingyuan Luo, Hoi Ki Wong, and Hiu To Tang verified the underlying data. Zhaoxiang Bian, Jingyuan Luo, and Jialing Zhang wrote the original draft. All authors contributed to the review and editing. Jingyuan Luo, Jialing Zhang, Hoi Ki Wong, and Hiu To Tang had access to the raw data. All authors had access to all data in the study, and the corresponding author had final responsibility for the decision to submit for publication.

## CONFLICT OF INTEREST

The authors declare no conflict of interest.

## Supporting information

Supplementary information.

Supplementary information.

## Data Availability

Individual, de‐identified patient data can be made available at the request of investigators who propose to use the data for methodologically sound research. Data will be made available 6 months after article publication, with no end date. Requests for de‐identified data should be made to the principal investigator.
