# Supplementary material for: Weekly symptom profiles of nonhospitalized individuals infected with SARS‐CoV‐2 during the Omicron outbreak in Hong Kong: A retrospective observational study from a telemedicine center
Source: J Med Virol. 2023 Jan 9;95(2):e28447. doi: 10.1002/jmv.28447 (PMC9880749; doi:10.1002/jmv.28447)
Supplement: Supplementary file 1 — Supplementary information. [file JMV-95-0-s001.pptx]

## Slide 1
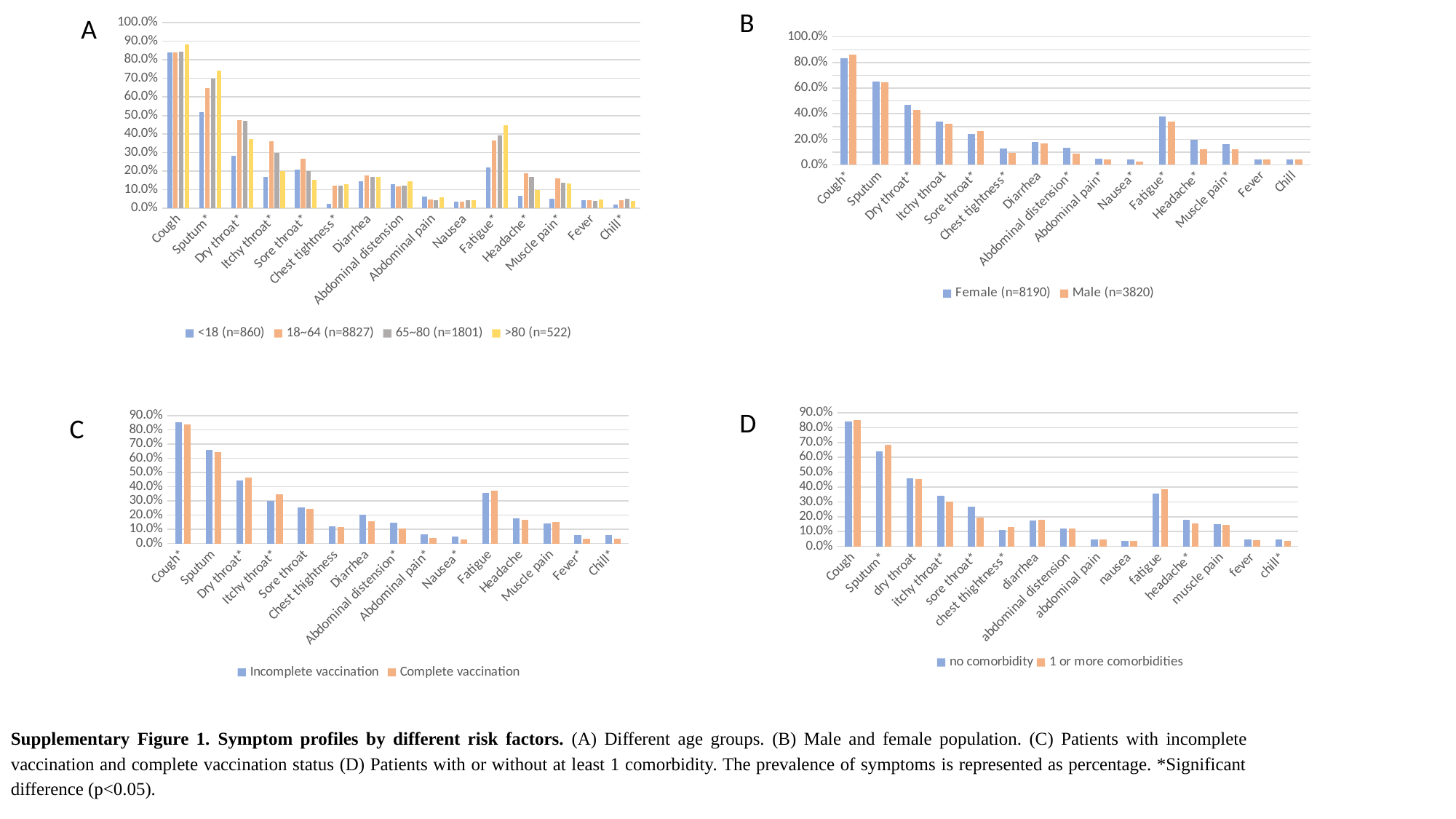

B
A
### Chart
| Category | <18 (n=860) | 18~64 (n=8827) | 65~80 (n=1801) | >80 (n=522) |
|---|---|---|---|---|
| Cough | 0.8418604651162791 | 0.8418488727767078 | 0.8456413103831205 | 0.8831417624521073 |
| Sputum* | 0.5186046511627908 | 0.6461991616630791 | 0.6996113270405331 | 0.7432950191570882 |
| Dry throat* | 0.28488372093023256 | 0.4755862694007023 | 0.4725152692948362 | 0.3735632183908046 |
| Itchy throat* | 0.1674418604651163 | 0.36082474226804123 | 0.29872293170460856 | 0.19923371647509577 |
| Sore throat* | 0.2069767441860465 | 0.268607680978815 | 0.20044419766796223 | 0.1532567049808429 |
| Chest tightness* | 0.02558139534883721 | 0.12371134020618557 | 0.12215435868961688 | 0.13026819923371646 |
| Diarrhea | 0.14651162790697675 | 0.17842981760507534 | 0.1693503609106052 | 0.17049808429118773 |
| Abdominal distension | 0.13023255813953488 | 0.11838676787130394 | 0.12215435868961688 | 0.14559386973180077 |
| Abdominal pain | 0.06279069767441861 | 0.04622181941769571 | 0.043309272626318715 | 0.05938697318007663 |
| Nausea | 0.037209302325581395 | 0.03625240738642801 | 0.0421987784564131 | 0.0421455938697318 |
| Fatigue* | 0.21976744186046512 | 0.3675087798799139 | 0.3947806774014436 | 0.446360153256705 |
| Headache* | 0.06627906976744186 | 0.18726634190551716 | 0.1693503609106052 | 0.09961685823754789 |
| Muscle pain* | 0.05232558139534884 | 0.16166307918885237 | 0.13603553581343697 | 0.13218390804597702 |
| Fever | 0.04302325581395349 | 0.04440919904837431 | 0.03886729594669628 | 0.04597701149425287 |
| Chill* | 0.022093023255813953 | 0.044522487821456895 | 0.04997223764575236 | 0.038314176245210725 |
### Chart
| Category | Female (n=8190) | Male (n=3820) |
|---|---|---|
| Cough* | 0.8356532356532357 | 0.862565445026178 |
| Sputum | 0.651037851037851 | 0.6455497382198953 |
| Dry throat* | 0.4711843711843711 | 0.4267015706806283 |
| Itchy throat | 0.3357753357753357 | 0.31963350785340316 |
| Sore throat* | 0.2424908424908425 | 0.2628272251308901 |
| Chest tightness* | 0.12808302808302807 | 0.09240837696335079 |
| Diarrhea | 0.17777777777777778 | 0.16727748691099475 |
| Abdominal distension* | 0.1354090354090354 | 0.09005235602094241 |
| Abdominal pain* | 0.05042735042735043 | 0.041361256544502616 |
| Nausea* | 0.04249084249084249 | 0.026701570680628273 |
| Fatigue* | 0.37704517704517704 | 0.3374345549738219 |
| Headache* | 0.19499389499389502 | 0.12303664921465969 |
| Muscle pain* | 0.16117216117216115 | 0.12198952879581151 |
| Fever | 0.04297924297924298 | 0.04476439790575916 |
| Chill | 0.042612942612942614 | 0.04528795811518325 |D
### Chart
| Category | no comorbidity | 1 or more comorbidities |
|---|---|---|
| Cough | 0.8419842312746385 | 0.8512856150104239 |
| Sputum* | 0.6385238720981166 | 0.6834607366226547 |
| dry throat | 0.4575120455540954 | 0.45552466990965956 |
| itchy throat* | 0.3393561103810775 | 0.30298818624044477 |
| sore throat* | 0.26631625054752517 | 0.1938846421125782 |
| chest thightness* | 0.11257117827420063 | 0.1299513551077137 |
| diarrhea | 0.1727989487516426 | 0.179638637943016 |
| abdominal distension | 0.12078405606657906 | 0.12161223071577484 |
| abdominal pain | 0.04774419623302672 | 0.046907574704656015 |
| nausea | 0.03745072273324573 | 0.037526059763724806 |
| fatigue | 0.35775295663600526 | 0.3856845031271717 |
| headache* | 0.17783618046430136 | 0.15392633773453787 |
| muscle pain | 0.15002190100744633 | 0.14454482279360667 |
| fever | 0.04456855015330705 | 0.0403057678943711 |
| chill* | 0.04577310556285589 | 0.03613620569840167 |
### Chart
| Category | Incomplete vaccination | Complete vaccination |
|---|---|---|
| Cough* | 0.8546743464776252 | 0.837913553895411 |
| Sputum | 0.6590607000443066 | 0.643409818569904 |
| Dry throat* | 0.44528134692069116 | 0.46411419423692635 |
| Itchy throat* | 0.30084182543198934 | 0.34858591248665954 |
| Sore throat | 0.25431989366415597 | 0.24573105656350053 |
| Chest thightness | 0.11896322552060257 | 0.1153948772678762 |
| Diarrhea | 0.20336730172795747 | 0.157017075773746 |
| Abdominal distension* | 0.1473194505981391 | 0.10512273212379936 |
| Abdominal pain* | 0.06424457244129375 | 0.03748665955176094 |
| Nausea* | 0.05161719096145326 | 0.028948772678762006 |
| Fatigue | 0.35445281346920693 | 0.3704642475987193 |
| Headache | 0.17700487372618523 | 0.16915688367129136 |
| Muscle pain | 0.14133805937084626 | 0.15314834578441835 |
| Fever* | 0.05759858218874612 | 0.03508537886872999 |
| Chill* | 0.062250775365529455 | 0.03215048025613661 |C
Supplementary Figure 1. Symptom profiles by different risk factors. (A) Different age groups. (B) Male and female population. (C) Patients with incomplete vaccination and complete vaccination status (D) Patients with or without at least 1 comorbidity. The prevalence of symptoms is represented as percentage. *Significant difference (p<0.05).

## Slide 2
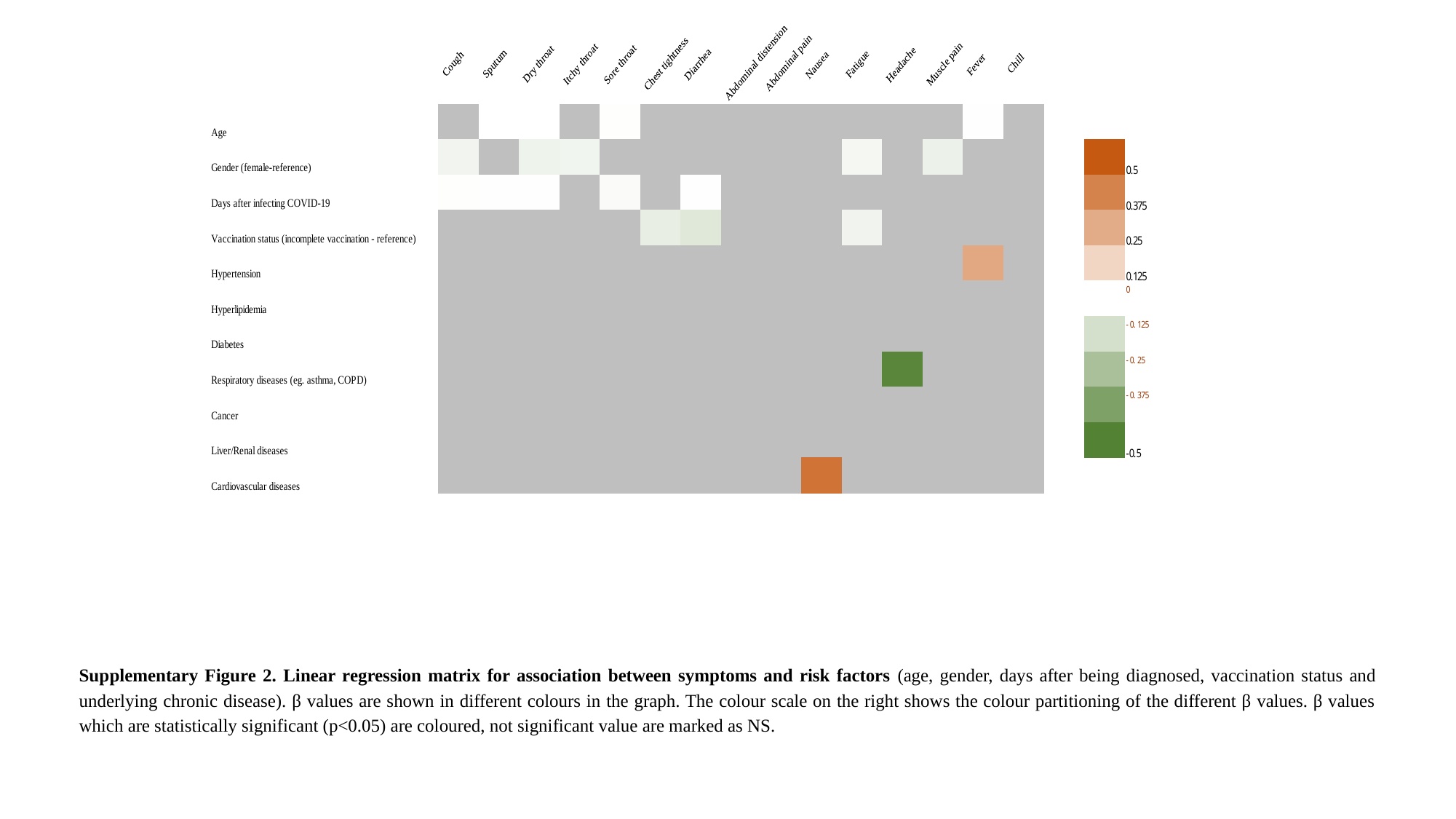

Supplementary Figure 2. Linear regression matrix for association between symptoms and risk factors (age, gender, days after being diagnosed, vaccination status and underlying chronic disease). β values are shown in different colours in the graph. The colour scale on the right shows the colour partitioning of the different β values. β values which are statistically significant (p<0.05) are coloured, not significant value are marked as NS.
