# Supplementary material for: Weekly symptom profiles of nonhospitalized individuals infected with SARS‐CoV‐2 during the Omicron outbreak in Hong Kong: A retrospective observational study from a telemedicine center
Source: J Med Virol. 2023 Jan 9;95(2):e28447. doi: 10.1002/jmv.28447 (PMC9880749; doi:10.1002/jmv.28447)
Supplement: Supplementary file 2 — Supplementary information. [file JMV-95-0-s002.docx]

**Supplementary Table 1. The definition of fixed symptoms and signs**

| **Symptom** | **Definition*** |
| --- | --- |
| Cough | Expel air suddenly and noisily from the lungs |
| Sputum | Increased production of mucus from respiratory tract |
| Itchy throat | A feeling of scratchy and sensitivity of the throat (easy to be aggravated by irritants) |
| Sore throat | A pain of the throat |
| Dry throat | A feeling of lacking liquid or moisture of the throat |
| Chest tightness | A squeezing, pressure, heaviness and tightness feeling in the chest |
| Diarrhea | Loose or watery and more-frequent bowel movements |
| Abdominal distension | A feeling of bloated in the abdominal area |
| Abdominal pain | A pain in the abdominal area |
| Nausea | A feeling of sickness with an urge to vomit |
| Fatigue | A feeling of weariness, tiredness, or lack of energy |
| Headache | A pain in the head |
| Muscle pain | Pain or tenderness in one or more muscles |
| Fever | Abnormally high body temperature: body temperature above the normal range of temperature screening device |
| Chills | A feeling of coldness |

Note: *The definitions were referred to the American Heritage Stedman's Medical Dictionary and Chinese Internal Medicine Text Book 7th edition (2004).

**Supplementary Table 2. Clinical characteristics of the patients who registered in the system within 7 days after tested positive, with or without at least one COVID-19 symptom**

| **Characteristic** | **Total**  **(N=4855)** | **Without COVID-19 symptoms**  **(N=137)** | **With COVID-19 symptoms**  **(N=4718)** | ***P* value*** |
| --- | --- | --- | --- | --- |
| **Age (years)** | 45 (34-61) | 45 (24.5-64) | 45 (34-61) | 0.066 |
| **Gender (N, %)** |  |  |  | 0.508 |
| Male | 1609 (33.1) | 49 (35.8) | 1560 (33.1) |  |
| Female | 3246 (66.9) | 88 (64.2) | 3158 (66.9) |  |
| **Dose of vaccination (N, %)** |  |  |  | 0.055 |
| Incomplete vaccination | 1954 (40.2) | 66 (48.2) | 1888 (40.0) |  |
| Complete vaccination | 2901 (59.8) | 71 (51.8) | 2830 (60.0) |  |
| **Days between last vaccination to infection** | 48 (21-171) | 40.5 (22.5-87.75) | 48 (20-173) | 0.753 |
| **Comorbidities (N, %)** |  |  |  | 0.309 |
| No comorbidity | 3835 (79.0) | 113 (82.5) | 3722 (78.9) |  |
| 1 or more comorbidities | 1020 (21.0) | 24 (17.5) | 996 (21.1) |  |
| **Subgroup of comorbidity (N, %)** |  |  |  |  |
| Hypertension | 564 (11.6) | 18 (13.1) | 546 (11.6) | 0.573 |
| Diabetes | 323 (6.7) | 10 (7.3) | 313 (6.6) | 0.758 |
| Hyperlipidemia | 354 (7.3) | 10 (7.3) | 344 (7.3) | 0.997 |
| Respiratory disease | 51 (1.1) | 0 (0.0) | 51 (1.1) | 0.221 |
| Cancer | 51 (1.1) | 1 (0.7) | 50 (1.1) | 0.709 |
| Cardiovascular disease | 127 (2.6) | 2 (1.5) | 125 (2.6) | 0.390 |
| Liver/Renal disease | 54 (1.1) | 4 (2.9) | 50 (1.1) | 0.041* |
| **Days after being diagnosed with COVID-19** | 4 (3-5) | 4 (2-5) | 4 (3-5) | 0.145 |
| **Days from first positive COVID-19 test to first negative test** | 7 (6-9) | 7 (8-9) | 7 (6-9) | 0.464 |

Note: Data are presented as medians (IQR) or number (%). *Significant level (p<0.05)

**Supplementary Table 3. Logistic regression analysis of the predictors of exhibiting at least one COVID-19 symptom for patients registered for online consultation within 7 days after tested positive**

| **Factors** | **Adjusted OR** | **95% CI** | ***P* value** |
| --- | --- | --- | --- |
| Age | 0.994 | 0.970 - 1.018 | 0.625 |
| Gender^1^ | 2.088 | 0.982 - 4.439 | 0.056 |
| Days after being diagnosed with COVID-19 | 1.052 | 0.847 - 1.307 | 0.649 |
| Vaccination status^2^ | 0.977 | 0.417 - 2.289 | 0.956 |
| Days between date of last vaccination to date of being diagnosed | 1.000 | 0.995 - 1.004 | 0.942 |
| Comorbidity^3^ | 1.276 | 0.472 - 3.452 | 0.631 |

Note: ^1^Female as reference. ^2^Incomplete vaccination as reference. ^3^No comorbidity as reference. *Significant association (p<0.05).
